# High-resolution contrast-enhanced vessel wall imaging in patients with suspected cerebral vasculitis: Prospective comparison of whole-brain 3D T1 SPACE versus 2D T1 black blood MRI at 3 Tesla

**DOI:** 10.1371/journal.pone.0213514

**Published:** 2019-03-08

**Authors:** Sebastian Eiden, Christopher Beck, Nils Venhoff, Samer Elsheikh, Gabriele Ihorst, Horst Urbach, Stephan Meckel

**Affiliations:** 1 Department of Neuroradiology, Faculty of Medicine, Medical Center–University of Freiburg, University of Freiburg, Freiburg, Germany; 2 Department of Nuclear Medicine, Faculty of Medicine, Medical Center–University of Freiburg, University of Freiburg, Freiburg, Germany; 3 Department of Rheumatology and Clinical Immunology, Faculty of Medicine, Medical Center—University of Freiburg, Freiburg, Germany; 4 Clinical Trials Unit, Faculty of Medicine and Medical Center—University of Freiburg, Freiburg, Germany; Vanderbilt University Medical Center, UNITED STATES

## Abstract

**Purpose:**

Vessel wall imaging (VWI) using T1 dark blood MRI can depict inflammation of intracranial arteries in patients with cerebral vasculitis. Recently, 3D VWI sequences were introduced at 3 Tesla. We aimed to compare 2D and 3D VWI for detection of intracranial vessel wall enhancement (VWE) in patients suspected of cerebral vasculitis.

**Methods:**

44 MRI scans of 39 patients were assessed that included bi-planar 2D T1 and whole-brain 3D T1 SPACE dark blood VWI pre and post contrast. Visibility and VWE were analyzed in 31 pre-specified intracranial artery segments. Additionally, leptomeningeal and parenchymal contrast enhancement was assessed.

**Results:**

Overall, more arterial segments were visualized with 3D VWI (p<0.0001). Detection of VWE showed fair agreement between 2D and 3D VWI (κ = 0.583). On segmental level, more VWE was detected in intradural ICA by 2D VWI (p<0.001) and in VA V4 segment by 3D VWI (p<0.05). 3D VWI showed more leptomeningeal (p<0.05) and parenchymal (p<0.01) contrast enhancement. In patients with positive diagnosis of cerebral vasculitis, sensitivity was of 67% (2D and 3D VWI) and specificity was 44% (2D VWI) and 48% (3D VWI); more VWE was seen in arteries distal to VA and ICA compared to non-vasculitic patients.

**Conclusion:**

2D and 3D VWI differed in the ability to detect VWE. Whole brain coverage with better evaluability of VAs and distal intracranial artery segments, and depiction of more parenchymal and leptomeningeal enhancement make 3D VWI more favorable. As VWE in arteries distal to VA and ICA may be used for discrimination of vasculitic and non-vasculitic patients, future increase in spatial resolution of 3D VWI sequences may be beneficial.

## Introduction

CNS vasculitis is a rare but potentially devastating disease that requires high diagnostic certainty and fast initiation of treatment. Particularly for primary angiitis of the CNS (PACNS) or secondary brain artery inflammation due to undiagnosed underlying systemic disease, the diagnosis remains challenging. For PACNS, biopsy of the leptomeninges and cortex remains the gold standard [1, 2]. However, several studies showed that sensitivity of invasive histopathologic diagnosis remains only 50–75% leading to many false negative results [3–5]. Standard MRI with MRA may depict large artery stenosis, parenchymal lesions, or parenchymal/leptomeningeal contrast-enhancement but lacks specificity to distinguish vasculitic stenosis [5–7]. The invasive technique digital subtraction angiography (DSA) detects intracranial vasculitis with a good sensitivity (60–90%), especially stenoses of the large and medium-sized intracranial vessels. Though, it misses most of the changes in smaller vessels or non-stenotic vasculitic lesions and may not differentiate between vasculitis and reversible cerebral vasoconstriction syndrome (RCVS) resulting in a very low specificity for intracranial vasculitis (6–30%) [5, 7].

Since the first description of vessel wall enhancement (VWE) in CNS vasculitis using high-resolution vessel wall imaging (VWI) MRI [8], many studies have demonstrated that VWI sequences can detect and discriminate vasculitic lesions from other vasculopathies such as atherosclerotic plaques or RCVS, which DSA often cannot discriminate [9–16]. In VWI MRI, 2D high-resolution T1w and T2w TSE sequences using blood-suppression are usually employed to visualize focal wall lesions and VWE [17, 18]. However, these sequences suffer from long imaging times since two orthogonal planes are needed for accurate lesion detection, and limited spatial coverage of whole brain arteries. The latter necessitates exact planning of scan area in case of visible stenotic lesions on MRA or parenchymal damage that may point into the direction of vessel wall pathology, but may the complete burden of vessel wall pathology may still be missed.

Recently, 3D VWI MRI sequences with variable refocusing flip angles were introduced which offer isotropic spatial resolutions, larger whole-brain coverage, and shorter acquisition times, at cost of slightly lower in-plane resolutions [19–21]. Furthermore, an improvement of the signal to noise in the vessel wall and of the contrast to noise vessel wall versus lumen in 3D versus 2D VWI sequences was described [22]. However, 3D sequences still lack validation for clinical use in patients with suspected intracranial large artery vasculitis or vasculopathy.

In this prospective study, we compared a 3D black blood VWI sequence with whole-brain coverage at 3 Tesla with 2D VWI in two orthogonal planes for the detection and characterization of VWE in a cohort of patients with clinical suspicion of intracranial vasculitis.

## Methods

### Patients

This single-center prospective observational study was approved by the ethical review board of the University of Freiburg Medical Center/Germany and was registered in the German Clinical Trial Register (ID: DRKS00009518). Patients were included between 07/2015 and 03/2016 if there was clinical suspicion of primary CNS vasculitis, large vessel CNS vasculopathy, or secondary involvement of intracranial arteries in systemic vasculitis. Written informed consent was obtained. All clinical diagnoses were confirmed by a senior rheumatologist who analyzed the diagnostic criteria for vasculitis S1 Table. Thereby, the rheumatologist had access to all clinical data including all neuroradiological imaging reports but was blinded to the readers‘ evaluation of the study.

### MRI protocol

All examinations were performed on a 3 Tesla MRI scanner (MAGNETOM Prisma, Siemens, Erlangen, Germany) with a 64-channel coil. The MRI protocol included a standard DWI sequence, a 3D multi-slab TOF MRA sequence of the arteries at the circle of Willis, followed by two orthogonally placed 2D T1 TIRM and one 3D T1 SPACE fatsat dark blood VWI sequences (MRI parameters are summarized in Table 1). Both 2D slabs had thickness of 31mm with limited vessel coverage. Thus, exact placement of these 2D slabs was planned individually by an experienced MRI radiologist aiming to cover possible intracranial stenosis or vessel luminal abnormalities as visualized on TOF MRA. Thereby, a suitable combination of two orthogonal planes was chosen for the affected vessel course (e.g. axial and coronal planes in MCA stenosis). If no obvious stenosis/luminal abnormality was present, standard axial and coronal planes were placed over the circle of Willis. The 3D VWI sequence had whole brain coverage including the upper neck (to the level of 4^th^ cervical vertebra). After intravenous administration of contrast media (Prohance, 0.5M) and a fixed delay of 4 min, first the 2D and then the 3D VWI sequence were repeated.

**Table 1:** 

	2D VWI—T1 TIRM dark blood	3D VWI—T1 SPACE fatsat dark blood
Slice orientation	axial, coronal, or sagittal	Sagittal
Field-of-view (FOV)	230 x 201 mm	230 x 230 mm
Matrix	512 x 410	256 x 265
Slice thickness	2 mm (10% gap)	0.9 mm
Spatial resolution	0.45 x 0.45 mm (in-plane)	0.9 mm (isotropic)
Number of slices	15	192
Scan time	4 min 6 sec	6 min 45 sec

MRI protocols of 2D and 3D VWI sequences at 3 Tesla.

### Image analysis

Images were evaluated on PACS workstation by two neuroradiologists: one junior reader and one senior reader (2 and 13 years of neuroimaging experience, respectively) that were blinded to clinical data. 2D and 3D VWI were analyzed in separate sessions after inspection of the TOF MRA and DWI images for potential vessel stenosis and ischemic changes. The following 31 pre-specified intracranial artery segments were assessed: ICA extradural (C1-C5 segment) and intradural (C6/7 segment); vertebral artery (VA) V3, V4, and V5; anterior cerebral artery (ACA) A1, A2, and A3; middle cerebral artery (MCA) M1, M2, M3, and M4; posterior cerebral artery P1, P2, and P3/4 (paired segments); and basilar artery (BA, non-paired). Both readers evaluated the image quality of each arterial segment in consensus using a semi-quantitative score from 0–2: 0 = not visualized or not evaluable (severe artifacts); 1 = evaluable with some artifacts or partially visualized, 2 = well evaluable and visualized). Presence of VWE was assessed separately by each reader and was further classified as concentric or eccentric and short- or long-segmental. Concentric VWE was defined if it involved the whole wall circumference and was uniformly distributed; eccentric VWE if either only part of whole wall was involved or if the enhancement of the wall circumference was non-uniformly distributed. VWE was defined as short-segmental if seen only on a single slice (2mm) of the 2D VWI or if revealed a longitudinal extension of ≤ 2 mm on 3D VWI; long-segmental involvement was defined if the extension of VWE as longer than 2 mm. Additionally, the presence of parenchymal or leptomeningeal contrast-enhancement was evaluated on both 2D and 3D VWI. In the case of differing results between the two readers, a consensus reading was performed.

### Statistical analysis

Statistical analyses were performed using SAS 9.2 (SAS Institute Inc., Cary, NC, USA). Agreement between two raters or between MRI VWI methods were assessed with Cohen’s kappa (κ) coefficient. We calculated simple κ; or weighted κ in case of more than two ordered categories. Results from two raters (or MRI VWI methods) were compared with McNemar’s test, i.e. a paired test comparing whether one rater more frequently produces positive results. In case of more than two categories, a generalization of McNemar’s test was applied (Bowker’s test for symmetry). When comparing the further characterization of VWE, only arterial segments that showed VWE on both VWI sequences, were evaluated. Sensitivity and specificity were calculated with confidence limits based on the exact binomial distribution. Results of VWE in vasculitic and non-vasculitic patients were compared with Fisher’s exact test.

## Results

### Patients and diagnoses

44 MRI examinations from 39 patients were analyzed (mean age; 52.5 years, range 14–77 years; 62% female). In 26% of MRI scans (12/44 MRI), cerebral ischemia was diagnosed on DWI. Patient details including diagnosis of CNS vasculitis or alternative neurological diagnosis are summarized in S2 Table. 44% of patients (17/39) had a diagnosis of extracranial vasculitis, systemic vasculitis, or other rheumatic disorder predisposing to secondary CNS vasculitis. 26% of patients (10/39) had a final diagnosis of intracranial CNS vasculitis, of these 50% (5/10) were histopathologically proved by brain biopsy or other specimen in case of systemic vasculitis (e.g. temporal artery in giant cell arteriitis). Of the patients with a diagnosis predisposing to secondary CNS vasculitis 41% (7/17) were diagnosed with CNS vasculitis. In 46% of MRI scans (21/44), an immunosuppressive medication was given prior to the MRI scan (median duration of intake, 60 days).

### Image quality and visualization of arterial segments

On 3D VWI, 86.4% (608/704) of paired arterial segments showed good image quality of the arterial wall, 11.2% (79/704) were partially evaluable, and 2.4% (17/704) were not evaluable. On the 2D VWI, 25.4% of paired arterial segments (179/704) were visualized with good image quality, 47.4% (334/704) were limitedly evaluable and 27.1% (191/704) were not evaluable S3 Table. Overall, all arterial segments were better visualized/evaluable on 3D VWI (p<0.0001). On 3D VWI, the extradural ICAs, VAs (V3-5 segments), and the peripheral artery segments (A2, A3, M2-4, and P2 and P3/4) were better visible (p<0.0001). In addition, the BA was also better evaluable on the 3D VWI (p<0.05). Arterial wall image quality was not different between 2D and 3D VWI for the proximal segments of the circle of Willis (intradural ICAs, ACA A1, MCA M1, and PCA P1).

### Detection and characterization of VWE:

**Fig 1:**
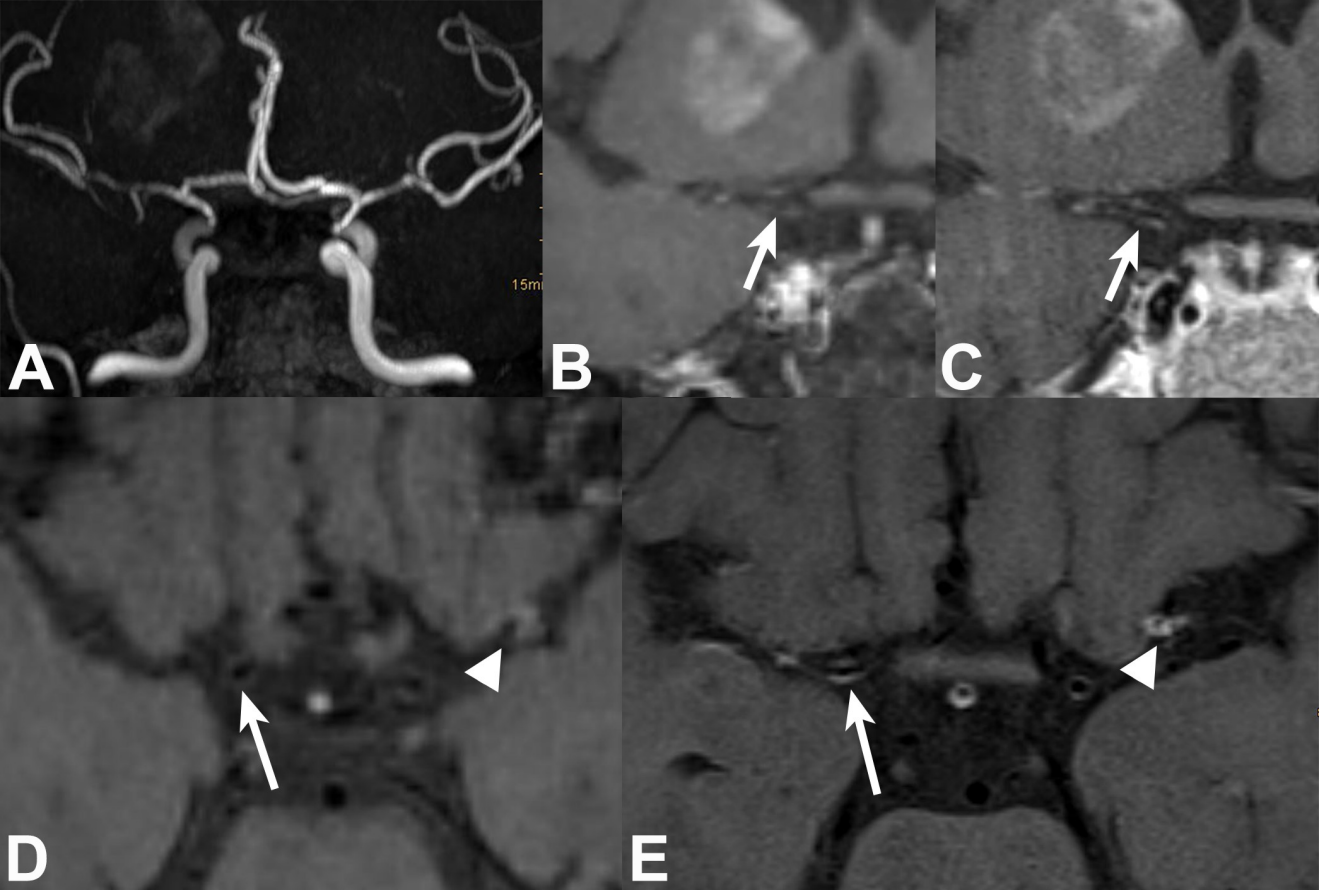
51-year-old female patient with neuroborreliosis, bilateral neuritis of 3rd nerve, and right MCA territory lenticulostriate infarct. TOF MRA (targeted MIP, **A**) shows bilateral medium-grade long-segmental stenosis of intradural ICAs and left MCA M1 segment, and high-grade right MCA stenosis. 2D and 3D VWI in coronal and axial views: Thin concentric VWE (*white arrow*s) of right supraclinoid ICA is barely visible on 3D (**B, D**) and clearly visible on 2D (**C, E**) VWI. Concentric VWE at left MCA bifurcation/proximal M2 (*white arrowheads*) is well visualized on both 2D (**E**) and 3D VWI (**D**).

**Fig 2:**
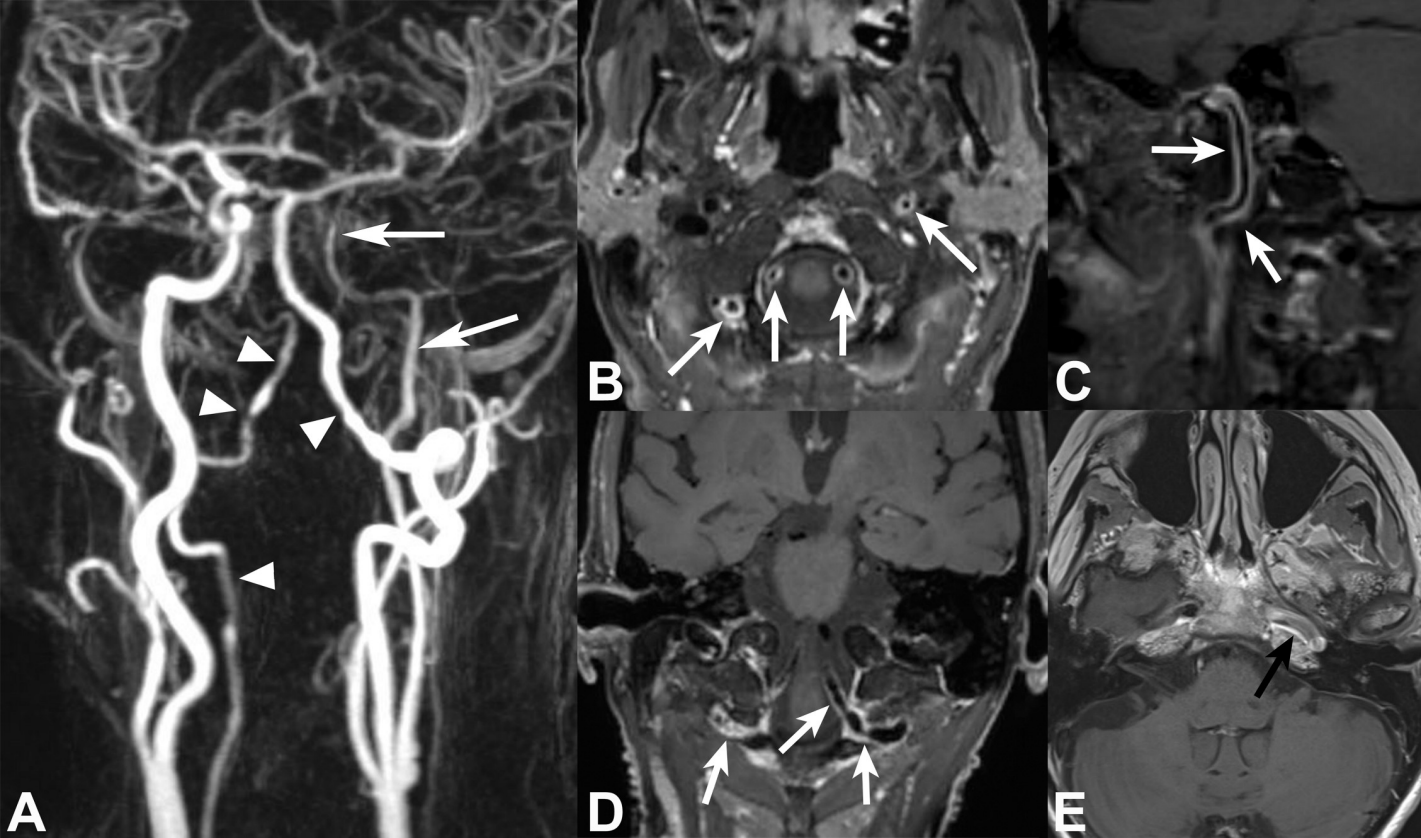
68-year female patient with giant cell arteriitis and left MCA territory infarcts. CE-MRA of supraaortic arteries (**A**) shows bilateral long segmental irregular VA stenosis involving right V3 and V4 as well as left V4 segments (*white arrowheads)* and long-segmental stenosis of left extra- and intracranial ICA (*white arrows*). 3D VWI with multiplanar views (**B-D**) depicts multiple areas of long-segmental concentric VWE in bilateral V3 and V4 segments and left cervical/petrosal ICA (*white arrows*). Due to limited coverage, 2D VWI shows only parts of the enhancing lesions in left petrosal ICA segment (*black arrow* in **E**).

Overall in all analyzed arterial segments, VWE detected by reader consensus showed a fair agreement (κ = 0.583) between 2D (77/1364 positive segments) and 3D VWI (78/1364 positive segments; Table 2). At individual segment level, VWE in the intradural ICAs was more frequently observed on 2D vs. 3D T1 VWI (p<0.001; Fig 1). Whereas in VA V4 segments, VWE was more frequently seen on 3D compared to 2D VWI (p<0.05; Fig 2). For the remaining arterial segments, VWE did not differ significantly between 2D and 3D VWI. When analyzed on a MRI scan level, any VWE was seen in 59.1% (26/44) on 2D vs. 56.8% (25/44) on 3D VWI with a good agreement (κ = 0.76). 2D and 3D VWI showed a fair agreement for characterizing short vs. long segmental VWE (κ = 0.58) and a poor agreement for concentric vs. eccentric type of VWE (κ = 0.05).

**Table 2:** 

Arterial Segment	2D VWI	3D VWI	P-value*
ICA, extradural	8.0% (7/88)	8.0% (7/88)	1.0000
ICA, intradural	23.9% (21/88)	8.0% (7/88)	0.0002
ACA, A1	3.4% (3/88)	2.3% (2/88)	0.3173
ACA, A2	1.1% (1/88)	3.4% (3/88)	0.1573
ACA, A3	0.0% (0/88)	1.1% (1/88)	0.3173
MCA, M1	6.8% (6/88)	4.5% (4/88)	0.1573
MCA, M2	12.5% (11/88)	10.2% (9/88)	0.1573
MCA, M3	0.0% (0/88)	1.1% (1/88)	0.3173
MCA, M4	0.0% (0/88)	1.1% (1/88)	0.3173
VA, V3	4.5% (4/88)	8.0% (7/88)	0.0833
VA, V4	19.3% (17/88)	33.0% (29/88)	0.0143
VA, V5	5.7% (5/88)	6.8% (6/88)	0.5637
Basilar artery	2.3% (1/44)	2.3% (1/44)	1.0000
PCA, P1	1.1% (1/88)	0.0% (0/88)	0.3173
PCA, P2	0.0% (0/88)	0.0% (0/88)	NaN
PCA, P3/4	0.0% (0/88)	0.0% (0/88)	NaN
All segments	5.6% (77/1364)	5.7% (78/1364)	0.8981

Frequencies of VWE on 2D and 3D VWI for all analyzed arterial segments. NaN = not a number.

*calculated with McNemar’s test.

Comparing only the arterial segments that were imaged and well evaluable (image quality score of 2) in both sequences (299/1364; S4 Table), VWE detected by reader consensus showed a fair agreement (κ = 0.592). Significantly more enhancement was observed on 2D VWI (26/299 positive segments) compared to 3D VWI (13/299 positive segments; p<0.001). Thereby, 2D VWI detected significantly more enhancement in the intradural ICA (19/72 vs. 7 /72 in 2D vs 3D VWI; p = 0.0005). 2D and 3D VWI showed a fair agreement for characterizing short vs. long segmental VWE (κ = 0.43). The calculation of agreement for the concentric vs. eccentric type of VWE was not possible.

### Inter rater agreement of VWE

Inter rater agreement for VWE was fair on 2D VWI (κ = 0.55) and good on 3D VWI (κ = 0.65). For characterizing short vs. long segmental VWE, interrater agreement was poor on 2D VWI (κ = 0.19) and fair on 3D VWI (κ = 0.47). For the differentiation of concentric vs. eccentric type of VWE, it was fair on 2D VWI (κ = 0.48) and poor on 3D VWI (κ = 0.26).

The senior reader found significantly more VWE on 3D (71/1364 positive segments) vs. 2D VWI (47/1364 positive segments; p<0.001; κ = 0.56). For the junior reader, VWE was not significantly different between 2D and 3D VWI (84 vs. 69 of 1364 segments, κ = 0.45).

### VWE in vasculitic versus non-vasculitic patients

In patients with a clinical diagnosis of CNS vasculitis, 10/15 of MRI scans showed any VWE on the 2D and 3D VWI, resulting in a sensitivity of 67% (95%-CI: 38–88%). In patients with diagnosis other than CNS vasculitis, VWE was seen in 16/29 on 2D and 15/29 on 3D VWI resulting in a specificity of 44% (95%-CI, 26–64%) for the 2D and of 48% (95%-CI, 29–67%) for the 3D sequence. From all patients with positive VWE, 38% (2D VWI) and 40% (3D VWI) were diagnosed with CNS vasculitis, 58% (2D VWI) and 56% (3D VWI) had a different neurological diagnosis, and 4% (both VWI sequences) did not have an alternative neurological diagnosis, respectively.

In all analyzed arteries (Table 3), significantly more VWE was encountered in vasculitic patients (2D VWI: 37/465; 3D VWI: 36/465) compared to non-vasculitic patients (2D VWI: 40/899; 3D VWI: 42/899) on both sequences (2D: p = 0.0093, 3D: p = 0.02630). Considering only arterial segments that were images and well evaluable arterial on both sequences (image quality score of 2), again significantly more VWE was found in vasculitic patients compared to non-vasculitic patients on both VWI sequences (2D VWI: p = 0.0163; 3D VWI: p = 0.0117; S5 Table). On individual segment level, VWE was significantly more present in the MCA M1 (p = 0.0213) and M2 (p = 0.0152) segments in vasculitic patients on 2D VWI and in the intradural ICA segments (p = 0.0429) on 3D VWI. Detailed characterization of VWE (short/long segmental and concentric/eccentric) showed not difference between vasculitic and non vasculitic patients.

**Table 3:** 

Arterial Segment	Vasculitic Patients		Non-vasculitic Patients	
	2D VWI	3D VWI	2D VWI	3D VWI
ICA, extradural	6.7%	10.0%	8.6%	7.4%
ICA, intradural	36.7%	16.7% #	17.2%	3.4% #
ACA, A1	6.7%	3.3%	1.7%	1.7%
ACA, A2	3.3%	6.7%	0.0%	1.7%
ACA, A3	0.0%	0.0%	0.0%	1.7%
MCA, M1	16.7% *	10.0%	1.7% *	1.7%
MCA, M2	26.7% *	20.0%	5.2% *	5.2%
MCA, M3	0.0%	0.0%	0.0%	1.7%
MCA, M4	0.0%	0.0%	0.0%	1.7%
VA, V3	6.7%	13.3%	3.4%	5.2%
VA, V4	16.7%	33.3%	20.7%	32.8%
VA, V5	0.0%	3.3%	8.6%	8.6%
Basilar artery	6.7%	6.7%	0.0%	0.0%
PCA, P1	0.0%	0.0%	1.7%	0.0%
PCA, P2	0.0%	0.0%	0.0%	0.0%
PCA, P3/4	0.0%	0.0%	0.0%	0.0%
All wo ICA/VA segments	5.4% *	4.1% **#**	1.0% *	1.5% **#**
ICA/VA segments only	13.3%	15.3%	11.7%	11.4%
All segments	8.0% *	7.7% #	4.4% *	4.7% #

Comparison of VWE in all analyzed arterial segments between vasculitic and non-vasculitic patients. All wo ICA/VA segments = all segments excluding ICA and VA segments. Significant difference of VWE between vasculitic and non-vasculitic patients is indicated by ^*^ on 2D and ^**#**^ on 3D VW.

A subgroup comparison of VWE was performed analyzing only the peripheral arterial segments after exclusion of the proximal extradural and large transdural segments (VA V3-V5 and intradural-/extradural ICA segments). In these segments, significantly more VWE was found in the vasculitic patients compared to the non vasculitic patients on both 2D (p<0.001) and 3D (p = 0.0204) VWI sequences. Whereas no significant difference in VWE was found in the excluded VA and ICA segments between the vasculitic and non-vasculitic patients.

Of the patients predisposing to secondary CNS vasculitis 47% (8/18) had intracranial VWE in both sequences.

### Brain parenchymal and leptomeningeal contrast-enhancement

Both readers detected contrast-enhancement in the brain parenchyma more frequently on 3D VWI (38.6%; 17/44 MRI scans) versus 2D VWI (25%; 15/44 MRI scans; p<0.05). The presence of leptomeningeal contrast-enhancement was also more commonly encountered on 3D with (22.7%; 10/44) versus 2D VWI (2.3%; 1/44; p<0.01).

## Discussion

In this study, we compared the detection and characterization of VWE of two commercially available 2D and 3D MRI VWI sequences at 3 T within a prospective cohort of patients that were examined under the clinical suspicion of intracranial vasculitis. 3D intracranial VWI sequences were recently introduced for high-field MRI since they offer high isotropic spatial resolutions with a good T1 contrast within clinically adequate scan times [21–23]. Our study showed that using 3D VWI with whole brain coverage both peripheral arterial segments (A2, P2, M2-4) and proximal segments, such as the extradural ICA, VA V3-5, and BA were better evaluable compared to a 2D VWI protocol acquired in two orthogonal planes. This was mainly attributed to the non-visualization of these vessel segments on both 2D planes due to limited spatial coverage. In addition, scan time was slightly shorter for 3D (6:45 min) vs. 2D (8:12 min for two orthogonal sequences) VWI protocol. For the detection of VWE, an overall fair agreement was found between 2D and 3D VWI which further improved when any enhancement per scan was regarded. At detailed analysis of arterial involved segments, VWE was more frequently observed on 2D VWI at the intradural ICA segment which may be attributed to the higher spatial resolution and signal-to-noise ratio of the 2D sequence. The latter may improve the visualization of faint VWE and the differentiation of VWE from closely enhancing venous structures such as the cavernous sinus (Fig 1). However, the higher in-plane resolution of 2D VWI sequences was at cost of a limited spatial coverage of the brain arteries compared to the 3D VWI sequence that covers the whole brain and upper cervical area. The latter was responsible for the better detection rate of VWE in the VA segments. A future direction to improve spatial resolution of 3D black blood imaging with whole brain coverage [22, 24–26] at adequate scan times may be the application of the recently introduced compressed sensing technology (CS-SPACE) [27].

**Fig 3:**
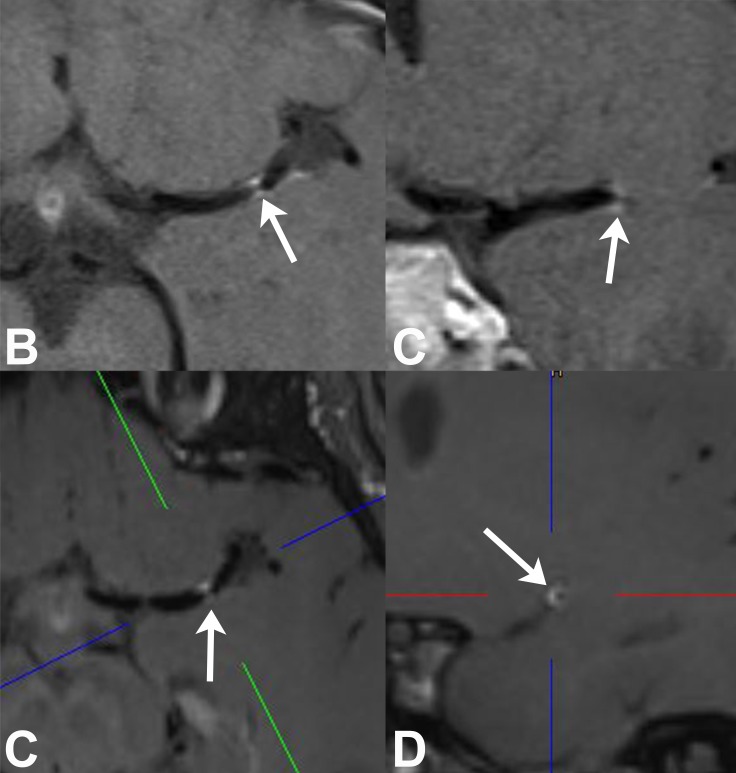
**39-year-old male patient with possible migraine and high-grade MCA stenosis:** 2D axial (**A**) and coronal (**B**) VWI reveals short segmental distal MCA VWE (*arrows*) that resembles a concentric appearance. 3D VWI with axial (**C**) and sagittal oblique (**D**) reformatted images: The latter image which is orientated perpendicular to MCA vessel course shows that there is obvious eccentric VWE (*arrow*) which is not obvious on standard 2D views.

Furthermore, the higher inter-rater agreement on 3D VWI was likely related to a superior analysis of the whole vessel wall circumference with 3D isotropic data (Fig 3). Such 3D visualization allows for a better discrimination between VWE of an artery and an enhancing vein being located adjacent to an artery [18]. Detailed characterization of VWE into concentric and eccentric/short and long segmental showed only a fair agreement between both sequences. This may again relate to differences in in-plane resolutions and 3D capabilities between both techniques. Both, high spatial resolution and 3D visualization are important to detect such fine differences in VWE that have been shown to be of diagnostic relevance: vasculitic lesions rather show a concentric VWE whereas atherosclerotic lesions mostly reveal an eccentric enhancement [9, 11, 12, 28–31].

Despite the technical progress of VWI and the implementation of clinical diagnostic guidelines, biopsy remains the gold standard of the diagnosis of cerebral vasculitis [1, 2, 32]. Though, several studies demonstrated a comparably low sensitivity and specificity of the brain biopsy with an elevated risk of complication compared to peripheral vessel biopsy [3–6, 17, 32]. MRI can help to improve sensitivity by localizing regions of active disease. *Salvarani et al*. revealed that around 8% of patients with cerebral vasculitis show leptomeningeal enhancement, which is a comparatively easily reachable location for a targeted biopsy [6]. Moreover, in small vessel vasculitis, patchy parenchymal or meningeal enhancement can be the only diagnostic hint apart from infarctions not restricted to a vascular territory [6, 33, 34]. In our study, 3D VWI detected more leptomeningeal and parenchymal enhancement likely related to its larger coverage and isotropic resolution providing additional important diagnostic information.

We also correlated the findings of VWE to the diagnosis of cerebral vasculitis obtained from all available clinical and imaging data. The overall sensitivity (67% for both sequences, respectively) and specificity (46% for both sequences) of any VWE was not very high. This may relate to the fact that the subtype of biopsy-proven PACNS may only involve small vessels and not show any VWE at the time of the diagnosis [15]. Moreover, many other conditions may lead to VWE like atherosclerotic plaques, intracranial artery dissections and also non pathological processes like the vasa vasorum [13, 35]. However, both VWI techniques showed more enhancing arterial segments in vasculitic patients, being significant on both sequences (p < 0.05). In particular, the proximal MCA segments (M1/M2) showed a higher involvement in patients with cerebral vasculitis which may be used as a potential discriminator since there is an emerging body of evidence that VWE may also occur in asymptomatic patients [19, 20].

In recent studies, 70% VWE was encountered in the VA or ICA where most atherosclerotic plaques which eccentric type of VWE are located as well as where the majority of vasa vasorum within intracranial arteries are found [35–39]. In a former study by *Takano et al*. with asymptomatic patients using 1.5T MRI, VWE occurred in 33% in the VA, in 14% in the intradural ICA and in 96% in the extradural ICA [40]. Whereas in the MCA and the BA, only 3.5% of VWE was seen in this asymptomatic cohort [40]. Moreover, recent studies by *Harteveld et al*. using 3D VWI sequences at 3T and 7T, demonstrated positive intracranial VWE in asymptomatic patients with the majority being located in the intradural VA and distal ICA segments [25, 41].

Along with these results, our subgroup analysis that excluded the ICA and VA segments showed an increased significance of VWE in the remaining cerebral arteries (ACA, MCA, BA, and PCA) for the diagnosis of cerebral vasculitis. On the other hand, these results underline the difficulty in interpreting the findings of VWE in the VA and ICA (Fig 2) which may require additional consideration of clinical data, as well as other imaging data on the vessel lumen (TOF- or CE-MRA) and the vessel wall lesion (T2-signal intensity) in order to discriminate inflammatory lesions from incidental findings [9, 12, 24].

Our study population included patients with intracranial artery dissections (n = 4) und large artery thrombosis (n = 1) which may cause local reactive VWE [9, 42]. This may be considered as a study limitation, yet it represents the nature of a real-world study approach with patients being scanned under the clinical suspicion of cerebral vasculitis or vasculopathy. Additionally 2D VWI was only acquired in two standard orthogonal planes to make the MRI acquisition non-dependent of the examiner, which limited the ability to evaluate all arterial segments perpendicularly to their vessel course. In the optimal case, the 2D VWI sequences should be placed perpendicular to a potential stenotic lesion seen on MRA imaging prior to acquisition of VWI, which appears as a further disadvantage of 2D VWI as it always requires the presence of a planning radiologist. Further limitations relate to the overall small number of patients, the fixed order of acquisition of the 2D VWI sequences before the 3D VWI after contrast media administration, and the fact that 49% of our patients were under immunosuppressive medication which has been described to reduce the findings of VWE in cerebral vasculitis [11, 43]. As only 4 patients received a follow up VWI in our study, the reduction of intracranial VWE under immunosuppressive medication could not be examined sufficiently. However this topic should be addressed more intensely in further studies.

## Conclusions

In summary, 3D VWI MRI in general did not differ in the ability to detect VWE compared to bi-planar 2D VWI. Mostly the larger coverage of the 3D sequence appears advantageous in detecting more VWE lesions, as well as meningeal or parenchymal contrast enhancement in areas not covered by the 2D sequence and omits the need for exact planning of scan area. In patients with cerebral vasculitis, overall more VWE was detected, and specifically in the proximal MCA segments on both VWI techniques. Whereas, VWE may also be seen in the distal VA and extradural ICA segments in asymptomatic or non-vasculitic patients. Some VWE lesions of the intradural ICAs were not seen on 3D compared to 2D VWI. As the latter VWI sequence has a higher in plane resolution, future increase in 3D spatial resolutions, e.g. with application of compressed sensing MRI technology may be of potential benefit.

## Supporting information

S1 Table Summary of criteria used by rheumatologist for diagnosis of vasculitis, rheumatoid disease, or other diseases predisposing to CNS vasculitis. (PDF)

S2 Table Summary of patients with clinical diagnosis of CNS vasculitis and diagnosis of systemic diseases predisposing to CNS vasculitis. CVID = common variable immune deficiency; GC = glucocortoid; L = lymph node; N = No; NA = not available; SAH = subarachnoid hemorrhage; SLE = systemic lupus erythematodes; Y = Yes.
(PDF)

S3 Table Consensus evaluation of vessel wall image quality for each arterial segment on 2D and 3D VWI. *indicates significant difference between 2D and 3D VWI.
(PDF)

S4 Table Frequencies of VWE on 2D and 3D VWI for all arterial segments that were rated well evaluable (image quality score of 2) on both sequences. The remaining segments showed an image quality score of less than 2 on either sequence and were determined not comparable (NC). NaN = not a number. *calculated with McNemar’s test.
(PDF)

S5 Table Comparison of VWE between vasculitic and non-vasculitic patients restricted only to arterial segments that were rated well evaluable (image quality score of 2) on both VWI sequences. The remaining segments showed an image quality score of less than 2 on either sequence and were determined not comparable (NC). Significant difference of VWE between vasculitic and non-vasculitic patients is indicated by * on 2D and ^#^ on 3D VWI.
(PDF)

## Abbreviatons

PACNS: primary angiitis of the CNS
RCVS: reversible cerebral vasoconstriction syndrome
VWE: vessel wall enhancement
VWI: vessel wall imaging
