# Supplementary material for: High-resolution contrast-enhanced vessel wall imaging in patients with suspected cerebral vasculitis: Prospective comparison of whole-brain 3D T1 SPACE versus 2D T1 black blood MRI at 3 Tesla
Source: PLoS One. 2019 Mar 8;14(3):e0213514. doi: 10.1371/journal.pone.0213514 (PMC6407784; doi:10.1371/journal.pone.0213514)
Supplement: S1 Table — (PDF) [file pone.0213514.s001.pdf]

Type of vasculitis, rheumatoid, or other disease predisposing to CNS vasculitis	Diagnostic criteria / reference
PACNS	1. Calabrese, L.H., et al., Primary angiitis of the central nervous system: diagnostic criteria and clinical approach. Cleve Clin J Med, 1992. 59(3): p. 293-306<br>2. Hajj-Ali, R.A., et al., Primary angiitis of the CNS. Lancet Neurol, 2011. 10(6): p. 561-72
SLE	Hochberg M.C., Updating the American College of Rheumatology revised criteria for the classification of systemic lupus erythematosus. Arthritis Rheum, 1997. 40: 1725
Giant cell arteritis	1. Hunder, G.G., et al., The American College of Rheumatology 1990 criteria for the classification of giant cell arteritis. Arthritis Rheum, 1999. 33:1122–1128<br>2. Jennette, J.C., et al., 2012 revised International Chapel Hill Consensus Conference Nomenclature of Vasculitides. Arthritis Rheum, 2013. 65:1–11
Large vessel vasculitis (aortitis)	Radiological diagnosis (aortic MR-angiography)
Neuroborreliosis	Antibodies in CSF analysis (lumbar puncture)
Antiphospholipid syndrome	Miyakis, S., et al., International consensus statement on an update of the classification criteria for definite antiphospholipid syndrome (APS). J Thromb Haemost, 2006. 4(2):295-306
Cryoglobulinemic vasculitis	Histopathology based diagnosis
Sarcoidosis	Hunninghake, G.W., et al., ATS/ERS/WASOG statement on sarcoidosis. American Thoracic Society/European Respiratory Society/World Association of Sarcoidosis and other Granulomatous Disorders. Sarcoidosis Vasc Diffuse Lung Dis, 1999. 16:149–173
Rheumatoid arthritis	Aletaha, D., et al., 2010 Rheumatoid arthritis classification criteria: an American College of Rheumatology/European League Against Rheumatism collaborative initiative. Arthritis Rheum, 2010. 62(9):2569-81
Polymyalgia rheumatica	1. Bird, H.A., et al., An evaluation of criteria for polymyalgia rheumatica. Annals of the Rheumatic Diseases, 1979. 38:434–439.<br>2. Dasgupta, B., et al., 2012 provisional classification criteria for polymyalgia rheumatica: a European League Against Rheumatism/American College of Rheumatology collaborative initiative. Ann Rheum Dis, 2012. 71:484–492
