# Supplementary material for: High-resolution contrast-enhanced vessel wall imaging in patients with suspected cerebral vasculitis: Prospective comparison of whole-brain 3D T1 SPACE versus 2D T1 black blood MRI at 3 Tesla
Source: PLoS One. 2019 Mar 8;14(3):e0213514. doi: 10.1371/journal.pone.0213514 (PMC6407784; doi:10.1371/journal.pone.0213514)
Supplement: S2 Table — CVID = common variable immune deficiency; GC = glucocortoid; L = lymph node; N = No; NA = not available; SAH = subarachnoid hemorrhage; SLE = systemic lupus erythematodes; Y = Yes. (PDF) [file pone.0213514.s002.pdf]

Patient (#)	Sex (M/F)	Age (years)	VWI MRI scans (#)	Immuno-suppressive medication Y/N (# days prior to MRI)	Immunosuppressive agent (cumulative dose prior to VWI)	Systemic disease predisposing to CNS vasculitis	Ischemia on DWI MRI Y/N	Biopsy Y/N (region)	Diagnosis of CNS vasculitis Y/N (alternative neurological diagnosis)
1	M	27	1	N		SLE	N	N	N (amaurosis fugax)
2	M	52	1	Y (18)	GC (3350 mg)		Y	N	N (thromboembolic stroke)
3	M	58	1	Y (5)	GC (500 mg)		N	N	N (cluster headache)
4	M	75	1	Y (306)	cyclophosphamide (6000mg), GC (4000mg)		Y	Y (brain)	Y
5	F	67	2	Y (110)	cyclophosphamide (1200mg), GC (4280mg)	giant cell arteriitis	N	Y (temporal artery)	Y
6	M	25	2	Y (195)	rituximab (4272 mg), cyclophosphamide (5900 mg), GC (1800 mg)	SLE	N	Y (nasal/LN)	Y
7	F	44	1	N			N	N	N (cryptogenic stroke)
8	M	34	1	N			N	N	N (possible RCVS)
9	F	29	1	N			N	N	N
10	F	76	1	N			N	N	N (cryptogenic SAH)
11	F	62	1	N		large vessel vasculitis (aortitis)	N	N	N
12	F	58	2	Y (4, 160)	GC (2000 mg)	large vessel vasculitis (thoraco-abdominal)	Y	Y (brain)	Y
13	F	68	1	N			Y	N	N (thromboembolic stroke)

14	F	51	3	Y (1, 15, 50)	GC (40mg)	neuroborreliosis w meningoradiculitis	Y	N	Y
15	F	71	1	Y (5)	GC (1150 mg)	giant cell arteriitis	Y	Y (temporal artery)	Y
16	F	40	1	N			N	N	N (intracranial artery dissection)
17	F	18	1	N		antiphospholipic syndrome	Y	N	N (microembolic infarcts)
18	F	70	1	N			Y	N	N (thromboembolic ICA occlusion)
19	M	77	1	N			N	N	N (cryptogenic SAH)
20	M	44	1	Y (5)	GC (350mg)		N	Y (temporal artery)	N (brain stem infarct, ICA dissection)
21	F	71	1	Y (12)	GC (260 mg)	cryoglobulinemic vasculitis / lymphoma	N	Y (skin)	N (TIAs)
22	M	28	1	N		sarcoidosis, Goodpasture syndrome	Y	Y (lung)	N (neurosarcoidosis)
23	M	47	1	N			N	N	N (thalamic infarct)
24	M	60	1	N			Y	N	N (VA dissection, PICA infarct)
25	F	39	1	Y (1825)	azathioprine (1825mg)		N	N	Y
26	F	44	1	N	NA	sarcoidosis	N	Y (bronchus)	N (cryptogenic SAH)
27	F	55	1	Y (60)	cyclophosphamide (13500 mg), GC (1725 mg)	SLE	N	N	Y
28	F	45	1	Y (120)	cyclosporine (NG)	SLE	N	N	N (recurrent hemiparesis)
29	M	61	1	N		antiphospholipic syndrome	N	N	N (thromboembolic infarcts)
30	F	52	1	Y (360)	azathioprine (36000 mg), cyclophosphamide (6000 mg), GC (1800 mg)	CVID, antiphospholipid syndrome, rheumatoid arthritis	N	N	Y

31	F	34	1	Y (180)	cyclophosphamide (13500 mg), GC (NA)		N	N	Y
32	M	54	1	N			N	N	N (carotidodynia)
33	F	15	1	Y (180)	GC (NG)	SLE	N	N	N (syncope)
34	M	74	1	N			N	Y (temporal artery)	N (TIA, macroangiopathy)
35	F	80	1	Y (1)	GC (250mg)		N	Y (temporal artery)	N (central retinal vein occlusion)
36	M	15	1	N			N	N	N (cryptogenic cerebellar infarcts)
37	M	77	1	Y (10)	GC (250mg)	polymyalgia rheumatica	N	N	N
38	M	46	1	N			N	N	N (bilateral VA dissection w infarcts)
39	F	63	1	Y (720)	etanercept (NA), GC (5400 mg)	rheumatoid arthritis	Y	Y (brain)	N
