# Supplementary material for: High-resolution contrast-enhanced vessel wall imaging in patients with suspected cerebral vasculitis: Prospective comparison of whole-brain 3D T1 SPACE versus 2D T1 black blood MRI at 3 Tesla
Source: PLoS One. 2019 Mar 8;14(3):e0213514. doi: 10.1371/journal.pone.0213514 (PMC6407784; doi:10.1371/journal.pone.0213514)
Supplement: S3 Table — *indicates significant difference between 2D and 3D VWI. (PDF) [file pone.0213514.s003.pdf]

| Arterial segment | Image quality scores | | | | | |
|---|---|---|---|---|---|---|
| | 2D VWI MRI | | | 3D VWI MRI | | |
| | 0 | 1 | 2 | 0 | 1 | 2 |
| ICA, extradural | 2.3%* | 97.7%* | 0.0%* | 2.3%* | 9.1%* | 88.6%* |
| ICA, intradural | 4.5% | 9.1% | 86.4% | 2.3% | 4.5% | 93.1% |
| ACA, A1 | 4.5% | 11.4% | 84.1% | 2.3% | 9.1% | 88.6% |
| ACA, A2 | 6.8%* | 88.6%* | 4.5%* | 2.3%* | 9.1%* | 88.6%* |
| ACA, A3 | 11.4%* | 88.6%* | 0.0%* | 2.3%* | 15.9%* | 81.8%* |
| MCA, M1 | 4.5% | 13.6% | 81.8% | 2.3% | 11.4% | 86.4% |
| MCA, M2 | 4.5%* | 93.2%* | 2.3%* | 2.3%* | 9.1%* | 88.6%* |
| MCA, M3 | 100%* | 0.0%* | 0.0%* | 2.3%* | 20.5%* | 77.3%* |
| MCA, M4 | 100%* | 0.0%* | 0.0%* | 2.3%* | 20.5%* | 77.3%* |
| VA, V3 | 29.5%* | 70.5%* | 0.0%* | 2.3%* | 9.1%* | 88.6%* |
| VA, V4 | 47.7%* | 52.3%* | 0.0%* | 2.3%* | 9.1%* | 88.6%* |
| VA, V5 | 27.3%* | 70.5%* | 2.3%* | 2.3%* | 9.1%* | 88.6%* |
| Basilar artery | 4.5%* | 34.1%* | 61.4%* | 2.3%* | 6.8%* | 90.1%* |
| PCA, P1 | 2.3% | 15.9% | 81.8% | 2.3% | 9.1% | 88.6% |
| PCA, P2 | 4.5%* | 93.2%* | 2.3%* | 2.3%* | 9.1%* | 88.6%* |
| PCA, P3/4 | 79.5%* | 20.5%* | 0.0%* | 2.3%* | 20.5%* | 77.3%* |
| All segments | 27.1%* | 47.4%* | 25.4%* | 2.4%* | 11.2%* | 86.4%* |
