# Supplementary material for: High-resolution contrast-enhanced vessel wall imaging in patients with suspected cerebral vasculitis: Prospective comparison of whole-brain 3D T1 SPACE versus 2D T1 black blood MRI at 3 Tesla
Source: PLoS One. 2019 Mar 8;14(3):e0213514. doi: 10.1371/journal.pone.0213514 (PMC6407784; doi:10.1371/journal.pone.0213514)
Supplement: S4 Table — The remaining segments showed an image quality score of less than 2 on either sequence and were determined not comparable (NC). NaN = not a number. *calculated with McNemar’s test. (PDF) [file pone.0213514.s004.pdf]

**Arterial segment**	**2D VWI**	**3D VWI**	**P-value***
ICA, extradural	NC	NC	NaN
ICA, intradural	26.4 % (19/72)	9.7 % (7/72)	0.0005
ACA, A1	3.0 % (2/66)	3.0 % (2/66)	1.0000
ACA, A2	0.0 % (0/4)	0.0 % (0/4)	NaN
ACA, A3	NC	NC	NaN
MCA, M1	6.5 % (4/62)	4.8 % (3/62)	0.3173
MCA, M2	0.0 % (0/2)	0.0 % (0/2)	NaN
MCA, M3	NC	NC	NaN
MCA, M4	NC	NC	NaN
VA, V3	NC	NC	NaN
VA, V4	NC	NC	NaN
VA, V5	0.0 % (0/2)	0.0 % (0/2)	NaN
Basilar artery	4.0 % (1/25)	4.0 % (1/25)	1.0000
PCA, P1	0.0 % (0/64)	0.0 % (0/64)	NaN
PCA, P2	0.0 % (0/2)	0.0 % (0/2)	NaN
PCA, P3/4	NC	NC	NaN
All segments	8.7 % (26/299)	4.3 % (13/299)	0.0008
