# Supplementary material for: High-resolution contrast-enhanced vessel wall imaging in patients with suspected cerebral vasculitis: Prospective comparison of whole-brain 3D T1 SPACE versus 2D T1 black blood MRI at 3 Tesla
Source: PLoS One. 2019 Mar 8;14(3):e0213514. doi: 10.1371/journal.pone.0213514 (PMC6407784; doi:10.1371/journal.pone.0213514)
Supplement: S5 Table — The remaining segments showed an image quality score of less than 2 on either sequence and were determined not comparable (NC). Significant difference of VWE between vasculitic and non-vasculitic patients is indicated by * on 2D and # on 3D VWI. (PDF) [file pone.0213514.s005.pdf]

Arterial segment	Vasculitic patients		Non-vasculitic patients	
	**2D VWI**	**3D VWI**	**2D VWI**	**3D VWI**
ICA, extradural	NC	NC	NC	NC
ICA, intradural	38.5 %	19.2 % #	19.6 %	4.3 % #
ACA, A1	5.0 %	5.0 %	2.2 %	2.2 %
ACA, A2	NC	0.0 %	NC	0.0 %
ACA, A3	NC	NC	NC	NC
MCA, M1	15.0 %	10.0 %	2.4 %	2.4 %
MCA, M2	NC	0.0 %	NC	0.0 %
MCA, M3	NC	NC	NC	NC
MCA, M4	NC	NC	NC	NC
VA, V3	NC	NC	NC	NC
VA, V4	NC	NC	NC	NC
VA, V5	0.0 %	NC	0.0 %	NC
Basilar artery	12.5 %	12.5 %	0.0 %	0.0 %
PCA, P1	0.0 %	0.0 %	0.0 %	0.0 %
PCA, P2	0.0 %	NC	0.0 %	NC
PCA, P3/4	NC	NC	NC	NC
All segments	15.3 % *	9.2 % #	6.0 % *	2.0 % #
